# Risk factors for pediatric invasive pneumococcal disease complicated by purulent meningitis

**DOI:** 10.3389/fped.2026.1714634

**Published:** 2026-04-10

**Authors:** Mi Yang, Zhenxing Liu, Ling Yang, Guangbo Li, Zhiqiang Zhuo, Dequan Su

**Affiliations:** 1 Department of Immunology and Rheumatology, Fudan University Affiliated Children's Hospital Xiamen Hospital (Xiamen Children's Hospital), Xiamen City, Fujian, China; 2 Department of Nephrology, Xiamen Hospital, Fudan University Affiliated Children's Hospital Xiamen Hospital (Xiamen Children's Hospital), Xiamen City, Fujian, China; 3 Department of Infection, Fudan University Affiliated Children's Hospital Xiamen Hospital (Xiamen Children's Hospital), Xiamen City, Fujian, China; 4 Xiamen Quality Control Center of Infectious Diseases, Xiamen City, Fujian, China

**Keywords:** children, invasive pneumococcal disease, NLR, PCT, purulent meningitis, risk factors

## Abstract

**Objective:**

To analyze the risk factors associated with invasive pneumococcal disease (IPD) complicated by purulent meningitis in children, with the goal of enhancing early diagnosis and treatment, preventing complications, and improving patient outcomes.

**Methods:**

The study involved 56 pediatric patients with IPD and admitted to our hospital from January 2016 and December 2024. Patients were stratified into two groups based on the presence or absence of purulent meningitis. Clinical characteristics and laboratory parameters were collected and analyzed using univariate and multivariate methods to identify risk factors. A risk prediction model based on logistic regression was developed, and its performance was assessed via the area under the receiver operating characteristic (ROC) curve.

**Results:**

The study cohort comprised 27 males and 29 females, including 13 patients with purulent meningitis and 43 without. Underlying diseases were present in 53.84% (7/13) of the purulent meningitis group compared to 4.65% (2/43) in the non-meningitic group (*P* < 0.001). Univariate analysis of laboratory indicators revealed significant intergroup differences in eight parameters (NLR, PCT, Alb, Na, Scr, D-Dimer, FDP, TT) (*P* < 0.05). Further, multivariate analysis identified PCT [odds ratio [OR] = 1.196, 95% confidence interval [CI]: 1.009–1.418, *P* = 0.039] and NLR (OR = 1.190, 95% CI: 1.014–1.395, *P* = 0.033) as independent risk factors for IPD complicated by purulent meningitis. The AUC for the model constructed with PCT > 4.215 ng/mL and NLR > 12.94 was 0.885, indicating that its predictive value for combined purulent meningitis is higher than that of the individual indicators, with sensitivity of 84.60% and specificity of 86%. Additionally, drug resistance analysis of 56 Streptococcus pneumoniae isolates revealed penicillin resistance rates of 73.21% (41/56) in meningitic strains vs. 60.71% (34/56) in non-meningitic strains, and ceftriaxone resistance rates of 28.57% (16/56) vs. 10.71% (6/56), respectively.

**Conclusion:**

Elevated PCT and NLR levels constitute independent risk factors for IPD complicated by purulent meningitis. The combined predictive model based on PCT > 4.215 ng/mL and NLR > 12.94 demonstrates robust clinical utility.

## 1 Introduction

*Streptococcus pneumoniae* (*S. pneumoniae*) is one of the most prevalent bacteria causing invasive infections in pediatric populations (1, 2). According to the Global Burden of Diseases, Injuries, and Risk Factors Study, infections due to *Streptococcus pneumoniae* were responsible for over 34,100 child deaths across 195 countries in 2016 (3). This data highlights the high incidence and mortality rates among children under 5 years of age and underscores the substantial burden imposed on healthcare systems worldwide (4, 5). Invasive pneumococcal disease (IPD) is notably linked to significant socioeconomic costs due to its high morbidity and mortality rates (6, 7). Previous studies have reported that 10% to 30% of invasive infections in children are complicated by meningitis, with these cases exhibiting a higher mortality rate (5–7). Furthermore, research indicates that 57% of Gram-negative invasive infections and 36% of confirmed Gram-positive invasive infections occur concurrently with meningitis (8). Notably, there are differences in antibiotic resistance between meningitic and non-meningitic strains of *Streptococcus pneumoniae*, with penicillin insensitivity rates of 69.5% and 35.9%, and the resistance rate in meningitic strains has been increasing over time (9). These findings suggest that IPD is more likely to be complicated by meningitis and that distinct bacterial resistance profiles exist. However, few studies have explored the risk factors for meningitis in children with IPD. Therefore, this retrospective single-center observational study aims to analyze the risk factors for meningitis and the patterns of bacterial resistance in pediatric patients with IPD by reviewing clinical records from our hospital.

## 2 Materials and methods

### 2.1 Study subjects

This was a retrospective single-center observational study. A total of 66 pediatric patients with IPD were identified at the Children's Hospital of Fudan University Xiamen Hospital (Xiamen Children's Hospital) between January 2016 and December 2024. Among these, 4 patients with organ dysfunction and 6 patients with incomplete clinical data were excluded, resulting in a final cohort of 56 patients for analysis. The cohort comprised 27 males and 29 females, corresponding to a male-to-female ratio of 0.93:1. Patients were stratified into two groups: 13 cases of complicated purulent meningitis and 43 cases of non-purulent meningitis. This study received approval from the Ethics Review Committee of Xiamen Children's Hospital and fulfilled the criteria for exemption from informed consent (Approval No. 20250605-1). All procedures were conducted in accordance with the Declaration of Helsinki.

### 2.2 Inclusion criteria

#### 2.2.1 Diagnostic criteria for pediatric IPD

(1) Patients aged 0–18 years; (2) Isolation of *Streptococcus pneumoniae* from sterile bodily sites such as blood, bone marrow, pleural effusion, ascites, or joint effusion (10).

#### 2.2.2 Diagnostic criteria for pneumococcal meningitis

(1) Clinical manifestations including fever, convulsions, headache, projectile vomiting, and varying degrees of consciousness impairment; in young infants, additional signs may include regurgitation of milk, irritability, abnormal crying, and fixed gaze. Meningeal irritation is evidenced by positive neck stiffness, Kernig's sign, and Brudzinski's sign; increased intracranial pressure may be indicated by a bulging anterior fontanelle or widened cranial sutures in infants and, in severe cases, cerebral herniation. (2) Cerebrospinal fluid analysis showing an elevated white blood cell count with neutrophilic predominance, significantly increased protein content, and markedly decreased glucose levels. (3) Positive cerebrospinal fluid culture for *S. pneumoniae* (11).

### 2.3 Exclusion criteria

Patients were excluded if they exhibited severe dysfunction of vital organs (e.g., heart, liver, kidneys), had incomplete clinical data, or were diagnosed and partially treated at other medical institutions.

### 2.4 Research methods

Demographic information and clinical data were collected, including the length of hospital stay, season of onset, white blood cell (WBC) count, neutrophil (NE) count, lymphocyte (LY) count, neutrophil-to-lymphocyte ratio (NLR), procalcitonin (PCT), hemoglobin (HGB), platelet count (PLT), C-reactive protein (CRP), erythrocyte sedimentation rate (ESR), alanine aminotransferase (ALT), aspartate aminotransferase (AST), albumin (ALb), lactate dehydrogenase (LDH), serum sodium (Na), serum creatinine (Scr), blood urea nitrogen (BUN), prothrombin time (PT), activated partial thromboplastin time (APTT), D-dimer (D-D), thrombin time (TT), and antimicrobial resistance profiles of *S. pneumoniae*.

### 2.5 Statistical methods

Analyses were performed using SPSS version 22.0. The normality of continuous variables was assessed using the Shapiro–Wilk test. Continuous variables with a normal distribution were presented as mean ± standard deviation and compared between groups using the t-test; non-normally distributed data were expressed as medians with interquartile ranges [M (IQR)] and compared using the Mann–Whitney U test. Categorical variables were summarized as counts (n) and percentages (%) and compared using the chi-square test. Collinearity diagnostics was performed with VIF (variance inflation factor). Significant variables in the univariate analysis (*P* < 0.05) and clinically meaningful variables were included in a multivariate linear regression. Logistic regression analysis was utilized for multivariate analyses, with *P* < 0.05 considered statistically significant. Receiver operating characteristic (ROC) curves were constructed to determine the optimal cutoff value, area under the curve (AUC), sensitivity, and specificity of each independent predictor and predictive model for combined purulent meningitis.

## 3 Results

### 3.1 Baseline demographic and clinical characteristics

**Figure 1:**
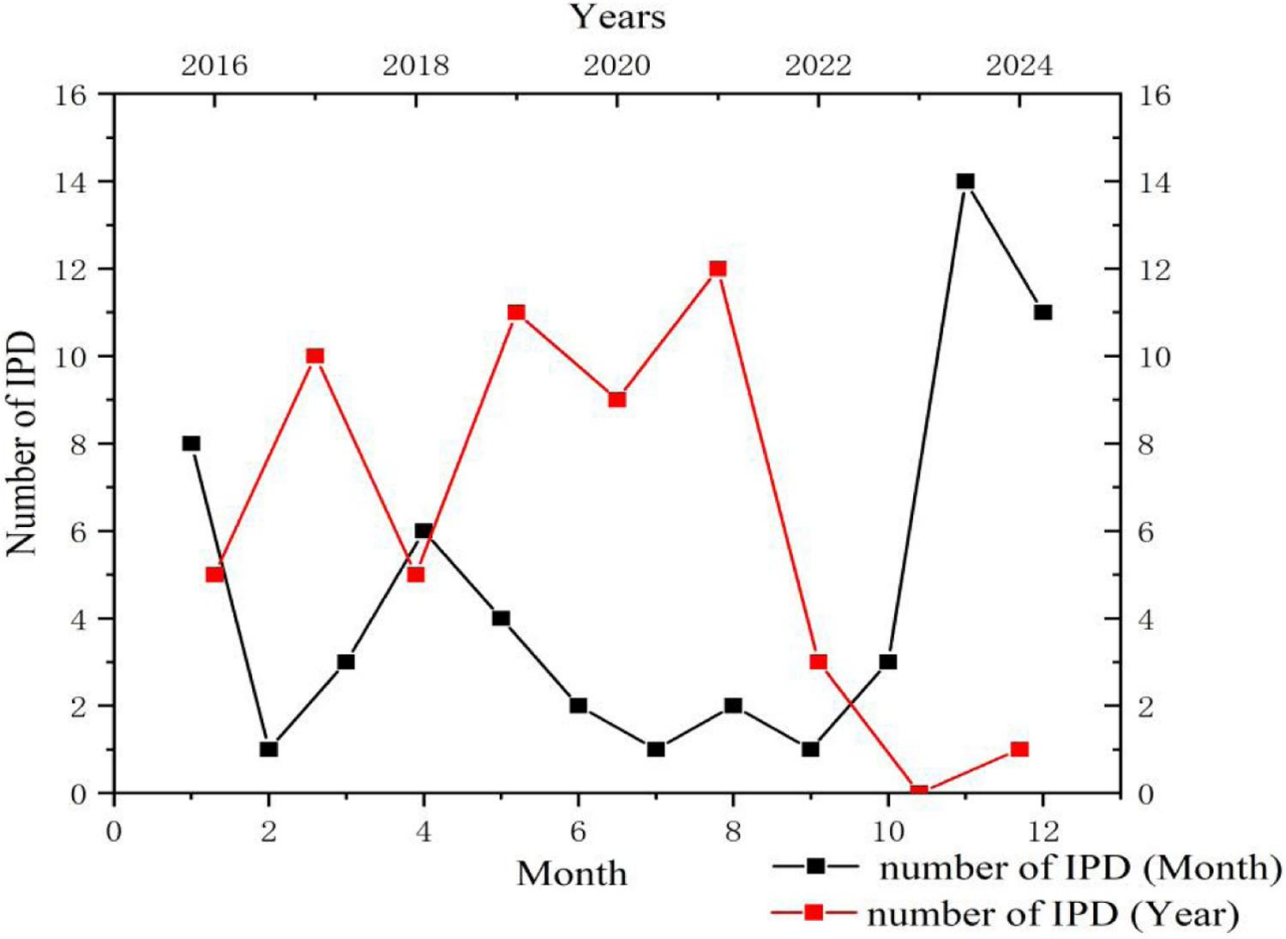
Distribution of invasive pneumococcal disease in our hospital from January 2016 to December 2024.

**Figure 2:**
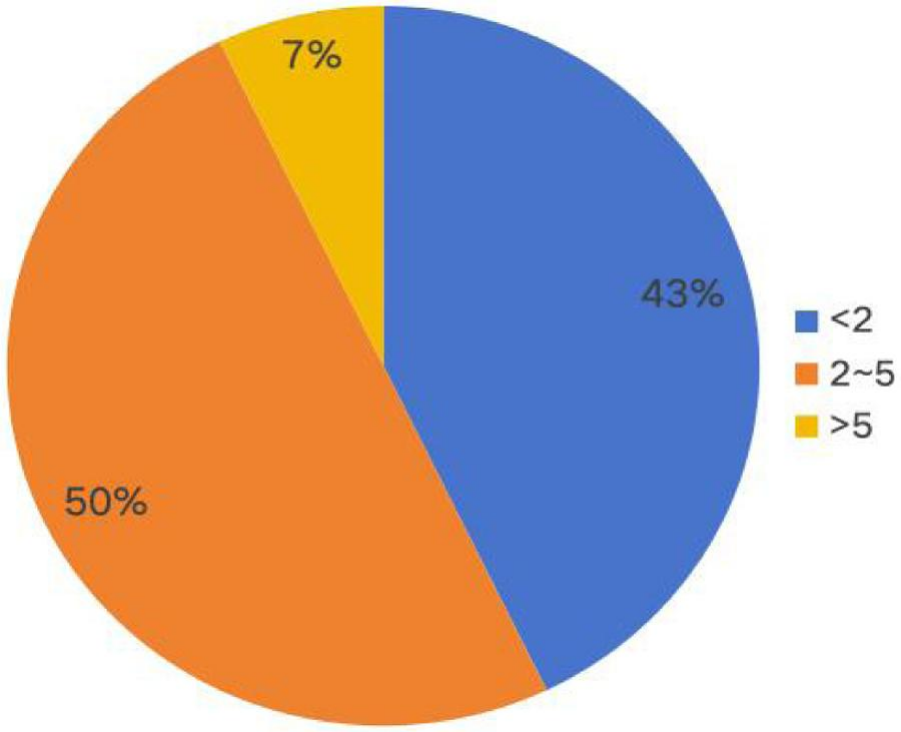
Age distribution of invasive pneumococcal disease.

A total of 56 community-acquired infection cases were included, comprising 27 males and 29 females, and the median age at onset was 30.50 months (IQR: 11.25–46.75 months). Notably, 42.90% of patients experienced disease onset before the age of 2 years, and 92.90% before 5 years. The highest incidence occurred between November and January, accounting for 58.93% (33/56) of total cases (Figures 1, 2). Patients were categorized into two groups based on the presence of purulent meningitis: a meningitis group (*n* = 13) and a non-meningitis group (*n* = 43). No statistically significant differences in gender, age at onset, season of onset, type of infection, or primary lesion were observed between the groups (*P* > 0.05). However, the proportion of patients with underlying diseases was significantly higher in the purulent meningitis group (*P* < 0.001). Four patients were co-infected with other pathogens: one with Respiratory Syncytial virus (RSV), one with both RSV and *Haemophilus influenzae*, one with Epstein–Barr virus (EBV), and one with Rhinovirus infection. The median length of hospital stay was 21.50 (10.25–38.00) days in the meningitis group, and median length of ICU stay was 3.50 (0.00–9.25) days, both significantly longer than those observed in the non-meningitis group (*P* < 0.05). Moreover, the meningitis group also had significantly higher mortality rate (23.08%; 3/13), than the non-meningitis group (*P* < 0.05) (Table 1).

**Table 1:** Baseline demographic and clinical characteristics.

Clinical characteristics		Meningitis group (*N* = 13) (%)	Nonmeningitis group (*N* = 43) (%)	x^2^/U	P
Age (months), M (P25, P75)		26 (11, 44)	38 (9.5, 59)	2.504	0.114
Sex				2.964	
	Male	4 (30.77)	23 (53.49)		0.151
	Female	9 (69.23)	20 (46.51)		
					
Season of onset					
	Spring	3 (23.08)	10 (23.26)	0.075	0.995
	Summer	1 (7.69)	4 (9.30)		
	Autumn	4 (30.77)	14 (32.56)		
	Winter	5 (38.46)	15 (34.88)		
Weight (P)					
	<P3	1 (7.69)	2 (4.65)	0.78	0.677
	P3-P97	12 (92.31)	39 (9.07)		
	>P97	0 (0.00)	2 (4.65)		
Place of residence					
	City	11 (84.62)	37 (86.05)	0.017	0.897
	Rural/suburban	2 (15.38)	6 (13.95)		
Primary foci of infection				2.208	0.137
	Respiratory tract	8 (61.54)	35 (81.40)		
	Others	5 (38.46)	8 (18.60)		
Underlying diseases/ Risk factors				18.222	<0.001
	Trauma or surgery	5 (38.46)	1 (2.33)		
	Immunocompromised	2 (15.38)	1 (2.33)		
	None	6 (46.15)	41 (95.35)		
Complicated with respiratory infections		8 (61.54)	35 (81.40)	2.208	0.137
Co-infection with other pathogens		0 (0.00)	4 (9.30)	0.277	0.598
Length of hospitalization (days)		21.5 (10.25, 38.00)	10 (7, 12)	7.501	0.016
Length of ICU stay (days)		3.5 (0, 9.25)	0 (0, 0)	15.746	<0.001
Death		3 (23.08)	1 (2.33)		0.035

### 3.2 Analysis of risk factors for IPD patients with complicated purulent meningitis

#### 3.2.1 Univariate analysis of laboratory parameters

WBC, NE, LY, NLR, CRP, Hb, PCT, AST, ALT, Alb, LDH, sodium, Na, Scr, BUN, D-dimer, FDP, APTT, TT, and PT were compared between patients with meningitis group and non-meningitis group. Statistically significant differences were identified in the NLR, PCT, Alb, Na, Scr, D-dimer, FDP, and TT (*P* < 0.05), whereas the remaining parameters did not show significant differences (*P* > 0.05) (Table 2).

**Table 2:** Univariate analysis of IPD patients with complicated purulent meningitis.

Laboratory indicators	Brain group	Amorphous brain group	P
WBC (*10^9/L)	19.65 ± 11.27	26.63 ± 10.67	0.622
NE (*10^9/L)	15.73 ± 9.07	20.04 ± 9.89	0.676
LY (*10^9/L)	2.69 ± 3.09	4.27 ± 2.83	0.639
NLR	10.72 ± 9.38	6.45 ± 4.73	0.003
CRP (mg/L)	121.73 ± 85.16	48.89 ± 53.09	0.072
HGB (g/L)	110.31 ± 18.69	114.83 ± 14.18	0.17
PCT (ng/mL)	22.19 ± 31.72	2.77 ± 4.46	<0.001
AST (U/L)	71.46 ± 103.7	40.56 ± 72.46	0.073
ALT (U/L)	21.15 ± 17.68	29.26 ± 79.48	0.504
Alb (g/L)	37.085 ± 8.21	42.070 ± 3.79	0.003
LDH (U/L)	477.46 ± 405.856	378.43 ± 307.191	0.09
Na (mmol/L)	133.43 ± 3.97	135.14 ± 2.62	0.027
BUN (mmol/L)	3.53 ± 1.57	3.30 ± 0.98	0.211
Scr (umol/L)	32.68 ± 26.79	27.68 ± 8.26	0.003
Fib (g/L)	4.76 ± 1.61	4.70 ± 1.45	0.953
D-dimer (mg/L)	7.81 ± 15.03	0.57 ± 0.98	0.002
FDP (ug/mL)	52.09 ± 91.02	4.57 ± 5.56	<0.001
APTT (s)	34.41 ± 9.29	34.06 ± 5.35	0.233
TT (s)	15.10 ± 3.56	14.35 ± 1.17	0.029
PT (s)	15.24 ± 3.12	15.01 ± 2.09	0.281

WBC, white blood cells; NE, neutrophil; LY, lymphocyte; NLR, ratio of neutrophil count to lymphocyte count; CRP, C-reactive protein; HGB, hemoglobin; PCT, procalcitonin; AST, glutamic-pyruvic transaminase; ALT, glutamic oxaloacetic transaminase; ALB, albumin; LDH, L-lactate dehydrogenase; Na, natrium; BUN, blood urea nitrogen; Scr, silicon controlled rectifier; Fib, fibrinogen; FDP, fibrinogen degradation products; APTT, activated partial thromboplastin time; TT, thrombin time; PT, prothrombin time.

#### 3.2.2 Multivariate logistic regression analysis

TT and FDP were removed because of multicollinearity in the multivariate analysis. The six laboratory indicators that reached statistically significant in the univariate analysis were subsequently entered into a binary logistic regression model. The multivariate analysis revealed that PCT [odds ratio [OR] = 1.196, 95% confidence interval [CI]: 1.009–1.418, *P* = 0.039] and NLR (OR = 1.190, 95% CI: 1.014–1.395, *P* = 0.033) served as independent risk factors for IPD patients with purulent meningitis (Table 3).

**Table 3:** Multivariate logistic analysis of risk factors for IPD complicated with purulent meningitis.

Laboratory indicators	B	S.E.	Wald	OR	95%CI		P
D-dimer (mg/L)	0.1*99*	0.355	0.316	1.221	0.609	2.445	0.574
PCT (ng/mL)	0.179	0.087	4.246	1.196	1.009	1.418	0.039
Na (mmol/L)	−0.231	0.173	1.78	0.794	0.565	1.114	0.182
Scr (umol/L)	−0.079	0.059	1.78	0.924	0.822	1.038	0.181
NLR	0.174	0.081	4.563	1.190	1.014	1.395	0.033
Alb (g/L)	−0.088	0.119	0.541	0.916	0.725	1.157	0.462

NLR, ratio of neutrophil count to lymphocyte count; PCT, procalcitonin; Na, natrium; Scr, silicon controlled rectifier; FDP, fibrinogen; TT, thrombin time; ALB, albumin.

#### 3.2.3 Predictive value and cutoff values

**Figure 3:**
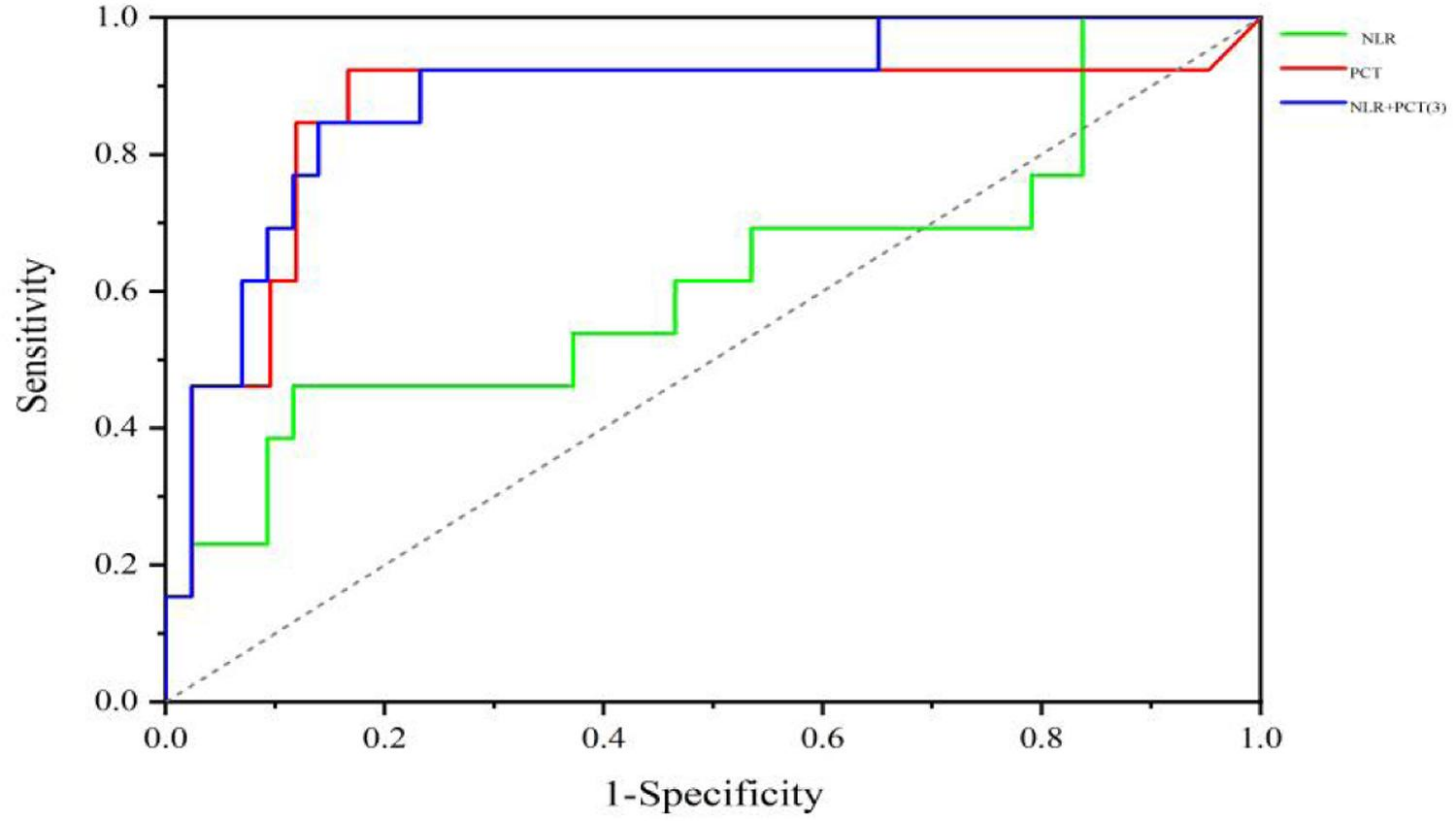
ROC curves of NLR, PCT, and NLR-PCT for predicting the presence of meningitis.

ROC curve analysis was conducted to determine the AUC, *p*-value, sensitivity, specificity, and optimal cutoff values—defined by the maximum Youden's index—for both PCT and NLR. The AUCs for PCT and NLR, were 0.863 and 0.606, while the AUC for the combination model of NLR and PCT were 0.885. The combination model demonstrated superior predictive value for *S. pneumoniae* purulent meningitis compared to individual markers, with sensitivity of 84.60% and specificity of 86.00% (Table 4 and Figure 3).

**Table 4:** Diagnostic value of NLR, PCT, and their combined model in predicting IPD complicated with purulent meningitis.

Laboratory indicators	Cut-off	AUC	SE	P	95%CI	Sensitivity	Specificity
NLR	12.94	0.606	0.102	0.251	0.406–0.806	46.20%	65.70%
PCT (ng/mL)	4.215	0.863	0.074	0.000	0.718–1.000	92.30%	83.30%
PCT+NLR	0.196	0.885	0.055	0.000	0.776–0.993	84.60%	86.00%

NLR, ratio of neutrophil count to lymphocyte count; PCT, procalcitonin.

### 3.3 Drug resistance analysis of *Streptococcus pneumoniae* strains

Fifty-six *S. pneumoniae* strains were isolated (strains obtained from different specimens from the same patient at the same time were considered identical). Among these, 53 cases had positive blood culture, 10 cases had positive cerebrospinal fluid culture, and 14 patients also had positive sputum cultures. One patient presented with both pyogenic arthritis and osteomyelitis, with *S. pneumoniae* detected in both blood and pus cultures. All strains underwent antimicrobial susceptibility testing. The prevalent rates of penicillin-resistant were 73.21% (41/56) and 60.71% (34/56) in meningitis and non-meningitis isolates, respectively. Similarly, 28.57% (16/56) of meningitis strains and 10.71% (6/56) of non-meningitis had resistance to ceftriaxone; 28.57% (16/56) of meningitis and 8.93% (5/56) of non-meningitis had intermediate resistance, respectively. The nonsusceptibility (resistance and intermediate susceptibility) rates to meropenem were 21.42% (12/56). Susceptibility rates to levofloxacin and erythromycin were 10.71% (6/56) and 1.79% (1/56), respectively. Notably, no vancomycin-resistant strains were detected (Table 5).

**Table 5:** Antimicrobial susceptibility of invasive pneumococcal isolates to common antibiotics.

Antimicrobial agent		S (%)	I (%)	R (%)
Penicillin (*n* = 56)				
	meningitis	10 (17.85)	5 (8.9)	41 (73.21)
	nonmeningitis	11 (19.64)	11 (19.64)	34 (60.71)
Ceftriaxone (*n* = 56)				
	meningitis	24 (42.86)	16 (28.57)	16 (28.57)
	nonmeningitis	45 (80.36)	5 (8.93)	6 (10.71)
Erythromycin (*n* = 56)		1 (1.79)	0 (0.00)	55 (98.21)
Levofloxacin (*n* = 56)		0 (0.00)	6 (10.71)	50 (89.29)
Vancomycin (*n* = 56)		56 (100)	0 (0.00)	0 (0.00)
Meropenem (*n* = 56)		44 (78.57)	10 (17.86)	2 (3.57)
SMZ (*n* = 53)		10 (17.86)	9 (16.07)	34 (60.71)

## 4 Discussion

In this study, we analyzed the clinical symptoms and laboratory indicators in 56 children with IPD and identified elevated PCT and NLR as independent risk factors for the development of purulent meningitis. The combination predictive model, using thresholds of PCT > 4.215 ng/mL and NLR > 12.94, demonstrated strong clinical performance in predicting purulent meningitis. Imaging and lumbar puncture examination was recommended in IPD patients with PCT > 4.215 ng/mL and NLR > 12.94, which is helpful for early diagnosis, early treatment, and improved prognosis. Additionally, meningitis strains exhibited higher resistance rates to ceftriaxone and penicillin compared to non-meningitic strains.

Our findings showed that sepsis was the most common manifestation of IPD, particularly among patients with oncological diseases or compromised immune function. Of the 56 children with IPD, 13 (23.21%) developed purulent meningitis. In a study by Cameron Burtond et al. (12), 12% of 93 children with IPD had concurrent purulent meningitis, while another study involving 377 children reported a rate of 29.8% (13). These results were consistent with our findings. Furthermore, our study indicated that children with underlying diseases were more prone to developing purulent meningitis; among the 13 children with purulent meningitis, 7 (53.84%) had comorbidities: 5 with tumors (and postoperative history) and 2 with immune deficiencies, suggesting that reduced immunity may facilitate bacterial invasion of the central nervous system. Previous studies reported mortality rates in children with IPD ranging from approximately 2.5% to 23.5% (13–15). In our cohort, the overall mortality rate was 7.14%, with 23.07% of patients with purulent meningitis, underscoring the need for clinical vigilance.

PCT is a propeptide glycoprotein produced by thyroid cells that rises significantly during the early stages of bacterial infection, making it a sensitive biomarker for diagnosing bacterial infections and sepsis, as well as for prognostication. Studies have shown that PCT levels correlate with the severity of pneumonia in patients with *S. pneumoniae* infection, and patients with bacteremia exhibited even higher levels of PCT, establishing PCT as a sensitive indicator for predicting *S. pneumoniae* bacteremia (16). A prospective cohort study of 1,821 febrile infants aged ≤60 days demonstrated that combination of PCT with urine analysis and neutrophil evaluation yielded a negative predictive value of 99.6% for bacterial meningitis (17, 18).

Our study reinforced the diagnostic value of PCT in IPD and highlight its potential role as a high-risk factor for bacterial meningitis. Moreover, the combination of PCT and NLR enhances predictive accuracy in these patients. NLR, calculated as the ratio of absolute neutrophil count to absolute lymphocyte count, reflects the body's immune response to various pathogenic agents. Previous studies have already suggested a link between NLR and sepsis outcomes, and investigated its utility in predicting the prognosis of bacterial meningitis (19–21). Our results add evidence supporting the use of NLR as a diagnostic tool in IPD-associated bacterial meningitis in children. The reason behind the increase in NLR among meningitis group is not fully understood. It is assumed to be associated with the body's innate (neutrophils) and adaptive (lymphocytes) immune responses. Elevated NLR levels, may indicate the host's immune response to streptococcus pneumoniae localized in the meninges.

This study has several limitations. First, our study was a single-center cohort study with a relatively small sample size, which may increase the risk of overfitting and impact the statistical power and limit the statistical stability and generalizability of the model. External validation was not performed, as the data were derived from a single institution. Consequently, the current model requires prospective evaluation in larger, multi-center cohorts prior to clinical application. Second, excluding some cases due to missing data may have introduce selection bias. Finally, the accurate histories of the pneumococcal vaccination status of these patients was not available due to the retrospective design of the study, which limits the interpretation of this result. However, our data found a significant reduction in the number of IPDs after 2023, which may be related to the implementation of free 13-valent pneumococcal vaccine in Xiamen from 2023. Future research should include multi-center, prospective, randomized controlled trials to confirm these findings.

## 5 Conclusion

Our study demonstrates that PCT (>4.215 ng/mL) and NLR (>12.94) are independent risk factors for developing purulent meningitis in children with invasive pneumococcal disease. These markers should be considered in the clinical diagnosis and management of affected patients.

## Data Availability

The original contributions presented in the study are included in the article/supplementary material, further inquiries can be directed to the corresponding author/s.
